# Exploring factors affecting nurse anxiety in Northwest Ethiopia: a multicenter study

**DOI:** 10.3389/fpsyt.2024.1434701

**Published:** 2024-10-02

**Authors:** Adane Misganaw, Mignot Hailu, Gashachew Bayleyegn, Mekidem Aderaw, Zeamanuel Anteneh Yigzaw, Tihtna Alemu, Lakew Asmare

**Affiliations:** ^1^ Department of Nursing, Debre Tabor Health Science College, Debre Tabor, Ethiopia; ^2^ School of Nursing, College of Medicine and Health Science, University of Gondar, Gondar, Ethiopia; ^3^ Department of Emergency and Critical Care Nursing, School of Nursing, College of Medicine and Health Science, University of Gondar, Gondar, Ethiopia; ^4^ Comprehensive Specialized Hospital, College of Medicine and Health Science, University of Gondar, Gondar, Ethiopia; ^5^ Department of Health Promotion and Behavioral Science, School of Public Health, College of Medicine and Health Sciences, Bahir Dar University, Bahir Dar, Ethiopia; ^6^ Department of Surgical Nursing, School of Nursing, College of Medicine and Health Science, University of Gondar, Gondar, Ethiopia; ^7^ Department of Epidemiology and Biostatistics, Institute of Public Health, College of Medicine and Health Sciences, University of Gondar, Gondar, Ethiopia

**Keywords:** anxiety, nurses, mental health, occupational stress, Ethiopia

## Abstract

**Introduction:**

Anxiety is a commonly reported mental health disorder that significantly contributes to the global burden of disease. However, proper counseling, problem-solving strategies, and promotion of healthy lifestyle practices have a positive effect on reducing nurse anxiety, but there is limited evidence in Ethiopia, particularly in this study area. Therefore, this study aimed to assess the prevalence and identify factors affecting nurse anxiety in Northwest Ethiopia’s comprehensive specialized hospitals.

**Methods:**

An institution-based cross-sectional study was conducted among 746 study participants from April 14 to May 20, 2023. A simple random sampling technique was used. An interviewer-administered questionnaire was used. Data were entered into EpiData version 4.6.0 and exported to STATA version 17 for analysis. In binary logistic regression, variables with a p-value of less than 0.25 were considered candidates for multivariable logistic regression. An association was declared at a p-value of less than 0.05 with a 95% confidence interval.

**Result:**

In this study, the prevalence of anxiety was 33.9%. Thus, being female [adjusted odds ratios (AOR) = 1.53, 95% CI = 1.08, 2.22], working in an emergency department (AOR = 3.65, 95% CI = 1.83, 7.28), working night duty shifts (AOR = 3.12, 95% CI = 2.19, 4.46), having conflict with coworkers (AOR = 1.7, 95% CI = 1.14, 2.51), and having poor social support (AOR = 2.13, 95% CI = 1.23, 3.69) were variables significantly associated with anxiety.

**Conclusion:**

This study revealed that one-third of nurses experienced anxiety, which is a critical indicator of mental health within this population. Important factors that were found to be significantly associated with anxiety include being female, working in the emergency department, having a conflict with coworkers, having poor social support, and working night duty shifts. These findings show the need for the implementation of counseling services and the adaptation of effective coping strategies for nurses working at comprehensive specialized hospitals. Understanding the impact of anxiety on nurses is important to design interventions aimed at improving their mental health and job satisfaction.

## Introduction

Anxiety is a psychological and physiological state characterized by cognitive, physical, emotional, and behavioral factors that can cause unpleasant feelings, anxious thoughts, and tension (1). Anxiety significantly contributes to the global burden of disease (2). According to a World Health Organization (WHO) report, 3.6% of the world population suffers from anxiety, and the total estimated number of people living with anxiety in the world is 264 million (3). Approximately 3.3% of people in Ethiopia are estimated to suffer from anxiety (4). The anxiety prevalence among healthcare professionals in Ethiopia was 26.8% (5). This prevalence is particularly relevant given the demanding nature of nursing work, which often includes high-stress environments and emotional labor (6). Nurses perform many responsibilities in hospitals such as caring for individuals and recovering optimal health and quality of life for their clients (7). Because of these activities and responsibilities, they often face enormous emotional pressure (8). Therefore, nurses frequently deal with unhappiness and become anxious in clinical settings (9, 10).

There are different causes of anxiety among nurses: challenging work environments, lack of human resources, and ongoing changes in the treatment and delivery of patient care (11). These factors affect professionals’ ability to perform their job by impairing their cognitive function, memory, and concentration, in addition to lack of physical exercise, smoking cigarettes, working in emergency departments, depression, night shift work, conflict with colleagues, and khat chewing (12, 13).

However, in recent years, several efforts have been made for nurses to reduce mental disorders, particularly anxiety through proper counseling regarding effective coping mechanisms and problem-solving strategies and the promotion of healthy lifestyle practices (6). Therefore, this study aimed to assess the prevalence and explore factors affecting nurse anxiety in Northwest Ethiopia’s comprehensive specialized hospitals. The findings of this study will provide important information for improving the quality of healthcare and the wellbeing of nursing professionals and treatment outcomes for patients.

## Methods

### Study design and setting

An institutional-based multicenter cross-sectional study was conducted from April 14 to May 20, 2023, in five comprehensive specialized hospitals found in Northwest Amhara: the University of Gondar Comprehensive Specialized Hospitals (UoGCSH), Debre Tabor Comprehensive Specialized Hospitals (DTCSH), Tibebe Ghion Comprehensive Specialized Hospitals (TGCSH), Felege Hiwot Comprehensive Specialized Hospitals (FHCSH), and Debre Markos Comprehensive Specialized Hospitals (DMCSH). These hospitals serve more than 20 million people. During the study period, a total of 1,610 nurses worked in these five hospitals.

### Population

The source population was all professional nurses who were working in Northwest Amhara comprehensive specialized hospitals. Permanently employed nurses working at least 6 months of relevant work experience in these hospitals during the data collection period were the study population. Nurses with known mental problems (based on their employment records) were excluded from the study.

### Sample size and sampling procedure

The sample size was determined using a single population proportion formula with a 95% confidence level, margin of error of 3% (0.03), 19.8% proportion of nurses experiencing anxiety from a previous study (6), and 10% non-response rate. Therefore, the final sample size was 746.

**Figure 1:**
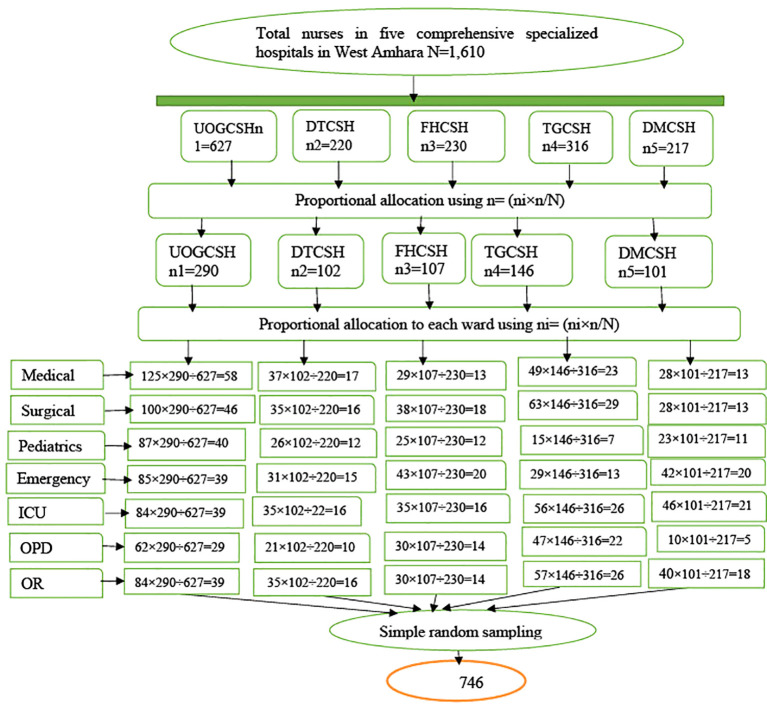
Schematic presentation of the sampling procedure used to select nurses working in Northwest Amhara comprehensive specialized hospitals, Ethiopia, 2023.

The total calculated sample size (746 nurses) was proportionally allocated to each hospital based on the lists of nurses from the hospital’s human resource management registration book: UoGCSH, 290; DTCSH, 102; FHCSH, 107; TGCSH, 146; DMCSH, 101. Finally, a simple random sampling (lottery method) technique was used (**Figure 1**).

### Operational definition of variables

#### Anxiety

Participants who scored below 10 on the Generalized Anxiety Disorder-7 (GAD-7) scale were considered to have no anxiety, and those who scored 10 and above were considered to have anxiety (14).

#### Depression

Participants who scored below 10 on the Patient Health Questionnaire-9 (PHQ-9) scale were considered to have no depression, and those who scored 10 and above were considered to have depression (15).

#### Stress

Study participants who scored ≥26 on the Workplace Stress Scale (WRSS) were considered to have work-related stress, and those who scored less than 26 had no work-related stress (16).

#### Social support

According to the social support scale, which is a brief three-item questionnaire (Oslo-3) that ranges from 3 to 14, those respondents who scored 3–8 were considered to have poor social support, those who scored 9–11 have moderate social support, and two who scored 12–14 have strong social support (17).

#### Current substance use

These were respondents who used substances (alcohol, khat, and cigarettes) in the last 3 months (18).

#### Ever substance use

These were respondents who used substances (alcohol, khat, and cigarettes) in their lifetime (18).

#### Physical exercise

A nurse was considered physically active if he/she exercised or performed any kind of sports activity including walking at least 150 min per week in a planned, structured, and repetitive manner (19).

### Data collection procedure and tools

Data were collected using a structured interviewer-administered questionnaire adapted from different literature settings (9, 10, 20, 21). First, the questionnaires were prepared in English, then translated into the local language Amharic, and translated back to English to check consistency by an independent translator. The questionnaires consisted of sociodemographic characteristics (sex, age, marital status, educational status, and monthly income), work-related and clinical factors (work experience, family history of mental illness, working department, job satisfaction, nurse–patient relationship, shift work, work overload, known chronic disease, position, lack of professional autonomy, and conflict with colleagues/coworkers), substance and behavioral-related factors, psychosocial factors (alcohol use, cigarette smoking, khat chewing, physical exercise, and leisure time activities), and psychosocial factors (depression, social support, and work-related stress). The questionnaire was pretested among 37 nurses at Woldia Comprehensive Specialized Hospital before the actual data collection period. Based on the findings of the pretest, minor modification was made to the questionnaires, and some statements were rephrased to make them easily understandable by study participants.

Anxiety was measured using the GAD-7 scale. The GAD-7 scale was reliable with Cronbach’s alpha of 0.92 and test–retest reliability (intra-class correlation = 0.83) with good validity (14). GAD-7 is a seven-item scale that measures anxiety. It was calculated by assigning scores “0” for not at all, “1” for several days, “2” for more than half of days, and “3” for nearly everyday responses for “how often have you been bothered by the seven listed symptoms of anxiety over the last 2 weeks?” Adding the scores, the GAD-7 scale ranged from 0 to 21. The total scores of measurements were interpreted as normal (0–4), mild (5–9), moderate (10–14), and severe (15–21). The cut-off score for detecting anxiety was ≥10. This scale was reliable and valid (14). In Ethiopia, it was validated with Cronbach’s alpha of 0.77 (22). It was widely used in Ethiopia among healthcare workers to measure anxiety (23, 24). Cronbach’s alpha reliability coefficient for GAD-7 in the current study was 0.89, demonstrating good internal consistency.

Depression was measured using the PHQ-9 scale, which comprises nine items that can be scored from 0 (not at all) to 3 (nearly every day). Scores of 0–4, 5–9, 10–15, and 20 represent cut points for normal, mild, moderate, and severe depression (15). The cut-off score for detecting depression was ≥10. It was validated in Ethiopia, and its Cronbach’s alpha was 0.78, indicating acceptable internal consistency (25). Cronbach’s alpha reliability coefficient for PHQ-9 in the current study was 0.81, indicating good internal consistency. Work-related stress was assessed using the WRSS composed of eight statements (16, 26). In terms of scoring, item numbers 6, 7, and 8 were reverse-scored with a 5-point Likert response format, ranging from never (scored 1) to very often (scored 5). Nurses who scored ≥26 on WRSS were considered to have work-related stress. The reliability of the tool was assessed on nurses, and its Cronbach’s alpha reliability coefficient was 0.79 (27). Cronbach’s alpha reliability coefficient for WRSS in the current study was 0.80, indicating good internal consistency. Also, social support was measured using the Oslo 3-item social support scale, and individuals who scored 3–8, 9–11, and 12–14 were considered to have poor, moderate, and strong social support, respectively (17). Cronbach’s alpha reliability coefficient for social support in the current study was 0.61.

Data were collected by five Bachelor of Science (BSc) psychiatric nurses and three Master of Science (MSc) supervisors after 2 days of training were provided. The training covered the goal, the purpose, the patient approach, confidentiality, and consent-taking technique. The collected data were reviewed for completeness and consistency by data collection supervisors and the principal investigator on a daily basis. Data cleaning and coding were performed before the analysis.

### Data processing and analysis

The data were coded and entered into EpiData version 4.6 for cleaning and exported to STATA version 17 for analysis. Descriptive statistics were computed and presented in the text, tables, and figure. Variables with p-values of less than 0.2 in the bivariate analysis were entered into the multivariable logistic regression model to determine the effect of each independent variable on the outcome variable. The variance inflation factor was equal to 1.48, which was less than 10, indicating that there was no multi-collinearity. The strength of the association was presented as the odds ratios and 95% confidence interval. A p-value of <0.05 in the multivariate analysis was considered significant. The result of model fitness using the Hosmer and Lemeshow test (p-value = 0.79) indicated that the model was fit.

## Results

### Sociodemographic characteristics

Among a total of 746 selected respondents, 732 (98.10%) participated in this study. The mean age of the respondents was 31.1 years with 3.97 (mean ± SD). More than half (54.10%) of the respondents were male. Concerning duty shifts, nearly two-thirds of respondents (62.16%) worked during the day shift. The majority (92.76%) of the participants did not have a family history of mental illness (**Table 1**).

**Table 1:** Sociodemographic, work-related, and clinical characteristics of nurses working in Northwest Ethiopia Comprehensive Specialized Hospitals, 2023 (n = 732).

Variables	Category	Frequency (n = 732)	Percentage (100%)
Sex	Male	396	54.10
	Female	336	45.90
Age (years)	18–24	3	0.41
	25–34	351	47.95
	35–44	277	37.84
	45–54	101	13.80
Marital status	Single	364	49.72
	Married	332	45.36
	Divorced	30	4.10
	Widowed	6	0.82
Educational status	Diploma nurse	85	11.61
	BSc nurse	618	84.43
	MSc and above	29	3.96
Monthly income in ETB	4,000–8,000	524	71.58
	8,001–10,154	208	28.42
Work experience (years)	≤4	67	9.15
	5–10	569	77.73
	11–14	75	10.25
	15–20	14	1.91
	21–30	7	0.96
Working department	Emergency	89	12.16
	ICU	120	16.39
	Medical ward	122	16.67
	OPD	86	11.75
	Surgical ward	119	16.26
	Pediatric ward	82	11.20
	Operation room	114	15.57
Family history of mental disorder	Yes	53	7.24
	No	679	92.76
Duty shift	Day	455	62.16
	Night	277	37.84
Professional autonomy	Yes	521	71.17
	No	211	28.83
Conflict with coworkers	Yes	201	27.46
	No	531	72.54
Known chronic disorder	Yes	45	6.15
	No	687	93.85

ICU, intensive care unit; OPD, outpatient department; ETB, Ethiopian Birr.

### Psychosocial, substance use, and behavioral characteristics

In this study, nearly half (49.18%) of participants reported using alcohol at least once during their lifetime. Currently, 93.03% of participants had never consumed alcohol. Regarding leisure time activities, most (82.92%) participants did not have leisure time activities, and 5.33% had them once or twice. A total of 34.02%, 13.39%, and 14.89% of respondents had poor social support, depression, and stress, respectively. Regarding the psychosocial characteristics of nurses, 34.02%, 13.39%, and 14.89% of respondents had poor social support, depression, and stress, respectively (**Table 2**).

**Table 2:** Psychosocial, substance use, and behavioral characteristics of nurses, 2023 (n = 732).

Variables	Category	Frequency (n = 732)	Percentage (100%)
Ever used tobacco	Yes	53	7.24
	No	679	92.76
Ever used alcohol	Yes	360	49.18
	No	372	50.82
Ever used khat	Yes	55	7.39
	No	677	92.61
Current tobacco use	Never	699	95.49
	Once/twice	29	3.96
	Daily	4	0.55
Current alcohol use	Never	384	52.46
	Once/twice	114	15.57
	Monthly	38	5.19
	Weekly	155	21.17
	Daily	41	5.60
Khat use	Never	681	93.03
	Once/twice	39	5.33
	Weekly	2	0.27
	Daily	10	1.37
Perform physical activity	Yes	193	26.37
	No	539	73.63
Leisure time activities	Yes	187	25.54
	No	545	74.46
Types of leisure time activities	Dancing	8	6.30
	Reading	65	51.18
	Walking	77	60.63
	Playing games	37	29.13
Social support	Poor	249	34.02
	Moderate	363	49.59
	Strong	120	16.39
Depression	No Depression	634	86.61
	Have depression	98	13.39
Stress	No stress	623	85.11
	Have stress	109	14.89

khat use = those who use khat in their lifetime.

### Magnitude of anxiety

The magnitude of anxiety among nurses was 33.90% with a 95% CI (30.5%, 37.4%) (**Table 3**).

**Table 3:** Indicators of anxiety measurement tool among nurses, 2023 (N = 732).

Component		Not at all	Several days	More than half the days	Nearly every day
Feeling nervous, anxious, or on edge		194 (26.5%)	347 (47.4%)	138 (18.85%)	53 (7.24%)
Not being able to stop or control worrying		318 (43.44%)	183 (25%)	174 (23.77%)	57 (7.79%)
Worrying too much about different things		293 (40.03%)	219 (29.92%)	149 (20.36%)	71 (9.7%)
Trouble relaxing		380 (51.91%)	136 (18.58%)	150 (20.49%)	66 (9.02%)
Being so restless that it is hard to sit still		302 (41.26%)	209 (28.55%)	159 (21.72%)	62 (8.47%)
Becoming easily annoyed or irritable		301 (41.12%)	223 (30.46%)	139 (18.99%)	69 (9.43%)
Feeling afraid, as if something awful may happen		334 (45.63%)	264 (36.07%)	94 (12.84%)	40 (5.46%)
Overall anxiety	No anxiety	66.10%			
	Have anxiety	33.90%			

### Factors associated with anxiety

In this model, the following variables were included in the multivariable analysis because their overall p-value in the bi-variable analysis was <0.25 (**Table 4**). Thus, the odds of anxiety were 1.53 times greater among female nurses than male nurses (AOR = 1.53, 95% CI = 1.07, 2.20). The number of nurses working in the emergency department was 3.65 times higher than that working in the outpatient department (AOR = 3.65, 95% CI = 1.83, 7.28). Additionally, nurses who were working night duty shifts were 3.12 times more likely to have anxiety than nurses who were working daytime shifts (AOR = 3.12, 95% CI = 2.19, 4.46). Respondents who experienced conflict with coworkers were 1.7 times more likely to develop anxiety than those who did not (AOR = 1.70, 95% CI = 1.14, 2.51), and those who had poor social support were 2.13 times more likely to develop anxiety than nurses who had strong social support (AOR = 2.13, 95% CI = 1.23, 3.69).

**Table 4:** Bivariable and multivariable analyses of factors associated with anxiety among nurses working in Northwest Ethiopia Comprehensive Specialized Hospitals, 2023 (n = 732).

Variables	Categories	Anxiety		OR with 95% CI	
		Yes N (%)	No N (%)	COR	AOR
Sex	Female	130 (17.8)	206 (28.1)	1.48 (1.09, 2.02)	1.53 (1.07, 2.20)*
	Male	118 (16.1)	278 (38)	1	1
Marital status	Single	124 (16.9)	240 (32.8)	1.13 (0.82, 1.55)	1.22 (0.83, 1.78)
	Married	104 (14.2)	228 (31.1)	1	1
	Divorced	16 (2.2)	14 (2)	2.21 (1.05, 4.68)	1.29 (0.51, 2.73)
	Widowed	4 (0.5)	2 (0.3)	3.87 (0.69, 21.43)	3.82 (0.46, 31.45)
Age	18–24	1 (0.1)	2 (0.3)	0.65 (0.06, 7.38)	0.95 (0.06, 14.9)
	25–34	112 (15.3)	239 (32.8)	0.61 (0.39, 0.95)	0.92 (0.41, 2.07)
	35–44	91 (12.4)	186 (25.4)	0.63 (0.39, 1.01)	0.95 (0.45, 2.03)
	45–54	44 (6.0)	57 (7.8)	1	1
Monthly income	4,000–8,000	168 (23)	356 (48.6)	0.75 (0.54, 1.05)	0.81 (0.55, 1.21)
	8,001–10,154	80 (10.9)	128 (17.5)	1	1
Working department	Emergency	54 (7.4)	35 (4.9)	3.63 (2.02, 6.51)	3.65 (1.83, 7.28)**
	ICU	55 (7.5)	65 (8.9)	1.99 (1.16, 3.41)	1.64 (0.86, 3.15)
	Medical ward	20 (2.7)	102 (13.9)	0.46 (0.24, 0.86)	0.51 (0.24, 1.05)
	Operation room	34 (4.6)	80 (10.9)	1.04 (0.56, 1.91)	0.98 (0.50, 1.91)
	Surgical ward	27 (3.7)	92 (12.6)	0.69 (0.38, 1.24)	0.62 (0.31, 1.25)
	Pediatrics ward	33 (4.5)	49 (6.7)	1.58 (0.87, 2.87)	1.54 (0.76, 3.10)
	OPD	25 (3.4)	61 (8.3)	1	1
Work experience	≤4	20 (2.7)	47 (6.4)	0.17 (0.03, 0.95)	0.13 (0.018, 1.04)
	5–10	185 (25.3)	384 (52.5)	0.19 (0.04, 1.00)	0.15 (0.02, 1.02)
	11–14	29 (4.0)	46 (6.3)	0.25 (0.05, 1.39)	0.17 (0.02, 1.12)
	15–20	9 (1.2)	5 (0.7)	0.72 (0.10, 05.17)	0.32 (0.04, 2.97)
	21–30	5 (0.7)	2 (0.3)	1	1
Duty shift	Night	143 (19.5)	134 (18.3)	3.56 (2.58, 4.90)	3.12 (2.19, 4.46)**
	Day	105 (14.3)	350 (47.8)	1	1
Professional autonomy	Yes	84 (11.5)	127 (17.3)	1.44 (1.03, 2.00)	1.31 (0.89, 1.94)
	No	164 (22.4)	357 (48.8)	1	1
Conflict with coworkers	Yes	90 (12.3)	111 (15.2)	1.91 (1.37, 2.67)	1.70 (1.14, 2.51)*
	No	158 (21.6)	373 (51.0)	1	1
Family history of mental illness	Yes	13 (1.8)	40 (5.5)	0.61 (0.32, 1.17)	0.56 (0.27, 1.17)
	No	235 (32.1)	444 (60.7)	1	1
Current khat use	Never	225 (30.7)	456 (62.3)	1	1
	Once/twice	14 (1.9)	25 (3.4)	1.13 (0.58, 2.23)	1.20 (0.56, 2.6)
	Weekly	1 (0.1)	1 (0.1)	2.03 (0.13, 32.6)	3.57 (0.08, 150)
	Daily	8 (1.1)	2 (0.3)	8.10 (1.70, 38.49)	3.18 (0.60, 16.7)
Social support	Poor	113 (15.4)	136 (18.6)	2.19 (1.37, 3.51)	2.13 (1.23, 3.69)*
	Moderate	102 (13.9)	261 (35.7)	1.03 (0.65, 1.63)	1.08 (0.63, 1.82)
	Strong	33 (4.5)	87 (11.9)	1	1

COR, crude odds ratio; AOR, adjusted odds ratio; ICU, intensive care unit. 1 = Reference.

*p-value < 0.05; **p-value < 0.001.

## Discussion

The study findings showed that 33.90% of nurses experienced anxiety, which is a critical indicator of mental health within this population. This prevalence aligns closely with findings from similar studies conducted in Kenya (35.6%) (28), South Tunisia (32.5%) (29), Italy (33.2%) (30), Iran (31.2%) (31), and China (37.3%) (32). The higher prevalence of anxiety in this study compared to that in previous studies in Addis Ababa (19.8%) (6) and Jimma (19.2%) (13) may be attributed to several factors, including differences in data collection methods and the ongoing socio-political challenges in northern Ethiopia, such as the impact of conflict and the COVID-19 pandemic (20, 21).

The result showed lower anxiety rates found in this study compared to studies conducted in Egypt (64.6%) (33) and South Africa (49.6%) (12) due to differences in inclusion and specific challenges faced by practitioners in different settings. For example, the Egyptian study included nurses with more than 1 year of experience, which may have affected the results. A significant proportion of participants reported poor social support (34.02%), depression (13.39%), and stress (14.89%). These factors are closely related, as poor social support can contribute to mental health issues, and mental illness in turn can lead to social isolation (34). The high prevalence of these problems underscores the need for interventions to improve social support and mental health in this population.

Important factors associated with anxiety include gender, working in emergency departments, working night shifts, and conflicts with coworkers. The finding is that female nurses are 1.53 times more likely to experience anxiety than their male counterparts. This finding is supported by studies conducted in Kenya (28) and Saudi Arabia (35). The possible reason may be biological factors such as differences in the brain structures involved in emotional regulation, which may explain the differences in the processing of negative information, the fluctuations of sex hormones that affect the anxious state of women, and genetic factors (36). Nurses working in the emergency department were nearly four times (3.65) more likely to develop anxiety than nurses working in the outpatient department. The finding of this study is supported by those of studies conducted in Iraq (37). This may be due to the need to deal with too many patients with inadequate numbers of nurses; an increase in the number of patients in emergency departments; and complex and complicated/severe cases, death of patients, and workload schedules in emergency departments. Nurses who were working night duty shifts were 3.12 times more likely to have anxiety than nurses who were working during daytime shifts. This finding is supported by a study conducted in Addis Ababa (6). This may be due to frequent and repeated night shift work, daytime sleepiness, lack of adequate sleep at night, fear of family separation, and difficulties in emotionally adapting to work at night. In this study, nurses who experienced conflict with coworkers were 1.70 times more likely to develop anxiety than nurses who did not have conflict with coworkers. This finding agrees with those of studies conducted among Chinese nurses (13, 32). The possible reason may be that conflict with colleagues increases the risk of experiencing anxiety episodes, which results from the secretion of increased cortisol and leads to damage of neurons in the hippocampus and amygdala, which are important structures of the brain that regulate mood and pleasure, resulting in anxiety (38), and nurses who had poor social support were 2.13 times more likely to develop anxiety than nurses who had strong social support. This finding is supported by studies conducted in South Africa (12) and Ethiopia (6). This may be due to loneliness, low socioeconomic status, death, separation from a loved one, and lack of family support. This may also be due to a lack of social relationships in health facilities, discrimination and stigma in the workplace, social disadvantage, physical violence, and poverty (39).

Furthermore, understanding the impact of anxiety on nurses may inform interventions aimed at improving their mental health and job satisfaction (40). Research in this area is critical for support programs and targeted interventions that can reduce anxiety and improve the overall work environment for clinical nurses.

## Strengths and limitations of the study

This is the first study of its kind in the study area, which provides updated information. Also, it may serve as the basis for future studies. However, the study was based on self-reported data; self-reports can lead to information bias, such as social desirability bias even though we tried to minimize it by reminding participants about confidentiality and anonymity during data collection.

## Conclusion

This study revealed that one-third of nurses experienced anxiety, which is a critical indicator of mental health within this population. Important factors that were found to be significantly associated with anxiety include being female, working in the emergency department, having a conflict with coworkers, having poor social support, and working night duty shifts. These findings show the need for the implementation of counseling services and the adaptation of effective coping strategies for nurses working at comprehensive specialized hospitals. Understanding the impact of anxiety on nurses is important to design interventions aimed at improving their mental health and job satisfaction. Research in this area is critical for support programs and targeted interventions that can reduce anxiety and improve the overall work environment for clinical nurses.

## Data Availability

The original contributions presented in the study are included in the article/supplementary material. Further inquiries can be directed to the corresponding author.
